# Remarks on a Peculiar Appearance Observed in the Gums of Consumptive Patients

**Published:** 1851-10

**Authors:** Theophilus Thompson

**Affiliations:** Physician to the Hospital for Consumption, &c.


### ARTICLE XVI.

*Remarks on a Peculiar Appearance observed in the Gums of Consumptive Patients.* By Theophilus Thompson, M. D., F. R. S., Physician to the Hospital for Consumption, &c.

The author after glancing at the risk of allowing the study of auscultation to supercede attention to general symptoms, refers to the discovery, communicated to the Society by the late Dr. Burton, of a blue line at the edge of the gums, as indicative of the presence of lead, and thence derives an encouragement to the examination of this part of the system, when there is reason to suspect any poisoned or morbid condition of the blood. He then presents the results of his observations in reference to this inquiry in cases of consumption, and avows his conviction of the frequent existence, in phthisical subjects of a mark at the reflected edge of the gums, deeper in color than the adjoining surface ; in some patients a mere streak on a raised border, in others, a margin more than a line in breadth, of a vermilion tint, inclining to lake ; the mark being most distinct around the lower incisors, but usually observable in both jaws, and often around the molar, but modified in its situation by the form of the mouth. The author has examined some hundred cases in the course of the investigation, and gives the analysis of 102, of whom he has full records. In forty of forty-eight women, the gingival margin is present ; and in fifty-four phthisical men, although in a few the line is so faint as to be open to question, there is only one in whom it can be considered decidedly absent. He has reasons for suspecting that the same condition of the system which produces this state of the gums tends also to produce clubbing of the fingers; but he considers that the change in the extremity of the fingers rarely occurs till some time after the streak is manifest in the gums. Of seventy-six patients, forty-five were found to have clubbed fingers; of these forty-five, only one had gums free from the characteristic margin; yet twenty of the seventy-six had marginated gums, but no expansion of the extremities of the fingers. The author discusses the effect of various modifying influences, such as hereditary tendency, catamenial disturbances, and habits as respects cleanliness, but cannot connect the presence of the symptoms in question with any of these circumstances; but he is of opinion that causes which irritate the mucous membrane tend to accelerate and increase the manifestation of the margin. He suggests this as an explanation of the more frequent absence of the line in women than in men, and dwells on its practical importance, as indicating, in such cases, the use of refrigerants, as preliminary to the introduction of tonic remedies. The author canvasses the question whether a similar line exists in any other disease; he allows that M. Fredericq may be correct in the opinion that certain changes in the gums occur towards the close of various chronic diseases, but he has never yet observed the peculiar margin described in this communication, without detecting other indications of consumption, although frequently only incipient. As respects prognosis in phthisis, he proposes the general rule, that cases in which the streak is observed early, or is broad or deep colored, tend to proceed more rapidly than those in which it is absent or slight; whilst freedom from the streak, even in the third stage, affords encouragement in treatment. In reference to diagnosis, the author believes, 1st. That the absence of the streak in men affected with inconclusive symptoms of phthisis, may incline us to a favorable interpretation of any such suspicious indications; but that in women, rather less weight is to be attributed to this negative sign. 2nd. That the presence of the sign in women is almost conclusive evidence of the presence of the tubercular element in the blood. The paper concludes with the remark, that the symptom therein described is one of many proofs that consumption is *not* exclusively a local disease, but rather a constitutional condition, requiring for its elucidation and treatment far more than an acquaintance, however exact, with the phenomena of auscultation.

[*London Lancet.*

